# Risk of anxiety and depressive disorders in patients with myocardial infarction: A nationwide population-based cohort study: Erratum

**DOI:** 10.1097/MD.0000000000006029

**Published:** 2017-01-20

In the article “Risk of anxiety and depressive disorders in patients with myocardial infarction: A nationwide population-based cohort study”,^[1]^ which appeared in Volume 95, Issue 34 of *Medicine*, Dr. Feng's affiliation was originally given incorrectly. The correct affiliation is as follows:

Graduate Institute of Medical Sciences and School of Nursing, National Defense Medical Center, Taipei City, Taiwan, Republic of China
